# Nanoparticle-Mediated Drug Delivery for Treatment of Ischemic Heart Disease

**DOI:** 10.3389/fbioe.2020.00687

**Published:** 2020-06-24

**Authors:** Chengming Fan, Jyotsna Joshi, Fan Li, Bing Xu, Mahmood Khan, Jinfu Yang, Wuqiang Zhu

**Affiliations:** ^1^Department of Cardiovascular Surgery, The Second Xiangya Hospital, Central South University, Changsha, China; ^2^Department of Cardiovascular Diseases, Mayo Clinic, Scottsdale, AZ, United States; ^3^Department of Emergency Medicine, The Ohio State University Wexner Medical Center, Columbus, OH, United States; ^4^Department of Physiology and Biomedical Engineering, Mayo Clinic, Rochester, MN, United States

**Keywords:** nanoparticles, controlled release, myocardial infarction, cardiac repair, drug delivery systems

## Abstract

The regenerative capacity of an adult cardiac tissue is insufficient to repair the massive loss of heart tissue, particularly cardiomyocytes (CMs), following ischemia or other catastrophic myocardial injuries. The delivery methods of therapeutics agents, such as small molecules, growth factors, exosomes, cells, and engineered tissues have significantly advanced in medical science. Furthermore, with the controlled release characteristics, nanoparticle (NP) systems carrying drugs are promising in enhancing the cardioprotective potential of drugs in patients with cardiac ischemic events. NPs can provide sustained exposure precisely to the infarcted heart via direct intramyocardial injection or intravenous injection with active targets. In this review, we present the recent advances and challenges of different types of NPs loaded with agents for the repair of myocardial infarcted heart tissue.

## Introduction

Ischemic heart diseases, caused by coronary artery obstruction, account for almost 80% of deaths from cardiovascular diseases (Lloyd-Jones et al., 2010). Traditional clinical approaches for myocardial infarction rely on surgical revascularization procedures, such as coronary stenting or coronary artery bypass grafts (CABG). Although the novel therapeutics using cells (especially stem cells) (Gao et al., 2013; Madigan and Atoui, 2018; Terashvili and Bosnjak, 2019), genes (Oggu et al., 2017), exosomes (Davidson and Yellon, 2018), and growth factors (Crafts et al., 2015; Reboucas et al., 2016) are emerging and have shown significant research outcomes, numerous challenges still exist in translating those technologies into clinical practice (Egwim et al., 2017).

**FIGURE 1:**
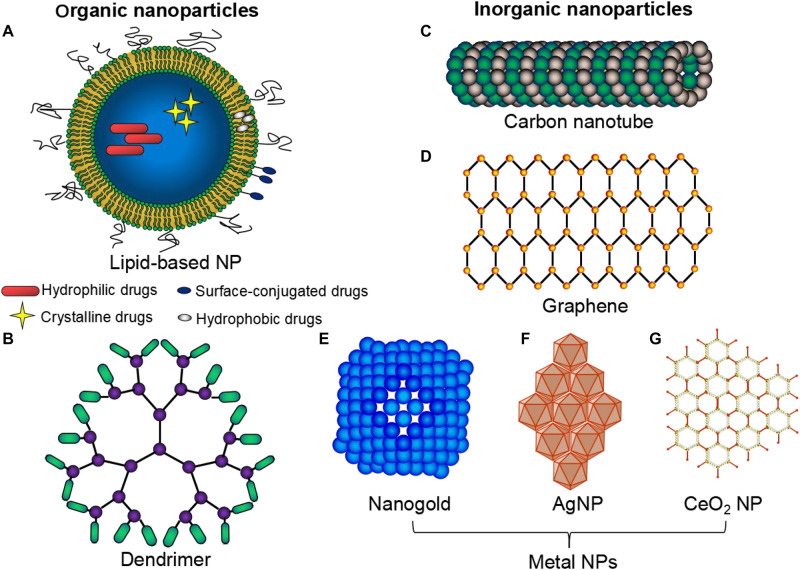
Types of nanoparticles commonly used in cardiac therapeutic studies. A wide variety of NPs: organic **(A,B)**, inorganic **(C–G)** and hybrid NPs are commonly used. Organic nanoparticles (NPs) are fabricated from proteins, carbohydrates, lipids **(A)**, and other organic compounds **(B)**, to a characteristic dimension. Inorganic NPs include carbon-based NPs e.g., carbon nanotubes **(C)**, graphene **(D)** and metal NPs e.g., gold **(E)**, silver **(F)**, and iron oxide **(G)**.

Nanoparticles have a long history. Faraday (1857) reported the synthesis of a colloidal Au NP solution for the first time. Similarly, Richard Feynman gave a talk in 1959 describing molecular machines built with atomic precision (Feynman, 1960). These were considered the very first reports on nanotechnology. Metal nanoparticles play a major role in the field of nanoparticle research (Jeevanandam et al., 2018). The 1950s and the 1960s saw the world turning its focus toward the use of nanoparticles in the field of drug delivery. Biological approaches for molecular nanotechnology were the first scientific conference held on the topic in the year 1996 (San Diego, CA, United States). Biological systems are organized at nanoscale dimensions and synthetic nanomaterials correlated in size with biological structures such as proteins, glycolipids, and DNA (Singh et al., 2011). Nanoparticles (NPs) are a type of nano-sized vesicles and can act as a sustained release delivery system of therapeutic agents and provide enhanced myocardial recovery in ischemic heart diseases (Binsalamah et al., 2011; Oduk et al., 2018). Nanoparticles can be classified either as organic (Figures 1A,B), inorganic (Figures 1C–G), or hybrid. Organic NPs usually show good biocompatibility, whereas inorganic NPs provide advantages in tailoring varied functions and properties (Vinhas et al., 2017). Organic nanoparticles are fabricated from proteins, carbohydrates, lipids, and other organic compounds to a characteristic dimension, such as a radius around 100 nm (Pan and Zhong, 2016), and are widely used NPs in cardiac therapy (Table 1). Inorganic NPs include carbon-based NPs, such as carbon nanotubes, buckyballs, and graphene, with remarkable features, strength and unique electrical properties (conducting, semiconducting, or insulating) (Vinhas et al., 2017). Besides, these inorganic NPs also include metal NPs, made of gold, silver, and iron oxide (Vinhas et al., 2017). Table 2 lists recent studies that used inorganic NPs for cardiac therapy. In the organic-inorganic hybrid nanoparticles, the organic functional groups combine the unique properties of the inorganic counterparts to confer efficient utility for various *in vivo* biomedical and clinical applications (Haque and Chowdhury, 2018). The use of hybrid nanoparticles for the slow release of drugs has been gaining great interest, particularly, to improve the selectivity and efficacy of the drugs by combining features of both organic and inorganic components in one nanoparticle system (Table 3).

**TABLE 1:** List of selective studies using organic nanoparticles for the delivery of therapeutics to repair infarcted myocardial tissue.

**Nanoparticles/size (nm)**	**Therapeutic agents**	**MI Model**	**Dose/administration route**	**Results**	**References**
Micelle/14.9 Liposome/101.5	None	Mouse, acute and chronic	50 μmol Gd/kg, intravenous	Micelles permeated the entire infarct area, Liposomes showed slower and restricted extravasation from the vasculature	Paulis et al., 2012
Micelles/90–100	ROS	Mouse, acute	3 mg, intramyocardial	Reduced infarct size and improved heart functions	Vong et al., 2018
PEG-PLGA/350	Liraglutide	Rat, chronic	380 μg, intramyocardial	Reduced infarct size, preserved wall thickness, stimulated angiogenesis, prevented cardiomyocyte apoptosis and improved heart functions	Qi Q. et al., 2017
PLGA/113	VEGF	Mouse, chronic	0.06, 2.6 or 0.6 pg, intramyocardial	Improved heart function, increased wall thickness, reduced infarct size and vasculogenesis	Oduk et al., 2018
PLGA/75	IGF1	Mouse, chronic	20 ng, intramyocardial	Prevented cardiomyocyte apoptosis, reduced infarct size, improved LV function and cardiac geometry	Chang et al., 2013
PK3/500	Nox2-siRNA	Mouse, chronic	5 ug/kg, intramyocardial	Improved fractional shortening	Somasuntharam et al., 2013
PK3/500	Nox2-miRNA	Mouse, chronic	5 ug/kg, intramyocardial	Improved fractional shortening and ejection fraction, reduced infarct size	Yang et al., 2017
Micelles/40	Nitroxyl radical	Dog, I/R	3 mg/kg, intravenous	Reduced infarct size and myocardial apoptosis	Asanuma et al., 2017
Micelles/34.7±14	CCR2	Mouse, acute	33 mg/kg, intravenous	No statistically significant improvements in cardiac function and infarct size	Wang et al., 2018
Silicon/100–200	siRNA, CCR2, MSCs	Mouse, chronic	25 mg/kg 1×10^5^ cells, intravenous	Improved LV remodeling amelioration and vascular density	Lu et al., 2015
Lipid/50	siRNA CRMP2	Mouse, chronic	70 μg/kg, intravenous	Reduced post-MI heart failure, mortality and fibrosis	Zhou J. et al., 2018
Lipid/<160	Cyclosporine A, ADSCs	Pig, chronic	2 mg/kg 4×10^7^ cells, intravenous	Improved heart function, reduced infarct size and cardiomyocyte apoptosis	Yin et al., 2014
Silicon/180	ERK1/2 inhibitor	Rat, acute	33 μg, intravenous	Reduced hypertrophy	Ferreira et al., 2017
PLGA/100	Cyclosporine A	Mouse, I/R	1 mg/kg, intravenous	Improved LV remodeling	Ikeda et al., 2016
PLGA/200	Irbesartan	Mouse, I/R	3 mg/kg, intravenous	Reduced infarct size and LV remodeling	Nakano et al., 2016
PLGA/160	Pitavastatin	Rat, I/R	1 mg/kg, intravenous	Reduced cardiomyocyte apoptosis	Mao et al., 2017
PLGA/160	Pitavastatin	Mouse, I/R	1 mg/kg, intravenous	Improved LV remodeling	Nagaoka et al., 2015
PLGA/160	Pitavastatin	Pig, I/R	8–32 mg/kg, intravenous	Improved LV remodeling, reduced infarct size and cardiomyocyte apoptosis	Ichimura et al., 2016
PEI/45	siRNA Icam1, Icam1, Vcam1, Sele and Selp	Mouse, chronic	1 mg/kg, intravenous	Reduced matrix-degrading protease activity	Sager et al., 2016
Lipid/110	Puerarin	Rat, chronic	50 mg/kg, intravenous	Reduced infarct size and oxidative stress	Dong et al., 2017
Lipid/84	Baicalin	Rat, chronic	10 mg/kg, intravenous	Reduced infarct size and oxidative stress	Zhang et al., 2016
Lipid/130	Schisandrin B	Rat, chronic	10 mg/kg, intravenous	Reduced infarct size	Shao et al., 2017
Lipid/<1000	Hemin	Mouse, chronic	2 mg/kg, intravenous	Improved infarct healing and repair	Ben-Mordechai et al., 2017
Lipid/105	Flavonoid	Rat, I/R	0.29 drug/lipid ratio, intravenous	Reduced infarct size	Tan et al., 2017
PEG-poly oxymethyl estyrene/40	2,2,6,6-tetramethyl piperidine-1-oxyl	Dog, I/R	3 mg/kg, intravenous	Reduced infarct size, apoptosis and ventricular fibrillation	Asanuma et al., 2017
Micellar/-	Rapamycin	Diabetic mouse, I/R	0.75 mg/kg/day, p.o. 10 weeks before I/R	Improved cardiac functions; reduced infarct size	Samidurai et al., 2017
Dendrimer/50	microRNA-1 inhibitor	Mouse, acute	15 μg, intravenous	Reduced cardiomyocyte apoptosis and infarct size	Xue et al., 2018

**TABLE 2:** List of selective studies using inorganic nanoparticles for the delivery of therapeutics to repair infarcted myocardial tissue.

**Nanoparticles/size (nm)**	**Therapeutic agents**	**MI Model**	**Dose/administration route**	**Results**	**References**
Graphene/30–40	VEGF	Rat, acute	300 μL, intramyocardial	Reduced infarct size, improved capillary density and cardiac performance	Paul et al., 2014
OPF/graphene oxide hydrogel	-	Rat, acute	100 μL, intramyocardial	Improved load-dependent ejection fraction/fractional shortening of heart function	Zhou J. et al., 2018
Graphene oxide complex/150	IL-4 pDNA	Mouse, acute	50 μL, intramyocardial	Attenuated inflammation, mitigated fibrosis and improved heart function	Han et al., 2018
Graphene oxide	Mesenchymal stem cell	Rat, I/R	One million MSCs	Improved the engraftment and therapeutic efficacy of MSCs, which promoted cardiac tissue repair and cardiac function	Park et al., 2015
Gold/10	PEG coated	Mouse, acute	100 μl/day, intravenous, 7 days	Decreased infarct size, improved systolic functions, inhibited cardiac fibrosis, no effect on apoptosis and hypertrophy	Tian et al., 2018
Copper/90–150	Cu	Rat, I/R	1 mg/kg/day, p.o.	Diminished oxidative stress, inflammatory cytokines and apoptosis, reduced infarct size	Sharma et al., 2018
Gold/50	Au	Rat, acute	(400 μg/kg/day) intravenous, 14 days	Improved myocardial injury	Ahmed et al., 2017
Cerium oxide/4–6	Ceria	Isoproterenol induced MI rat	(0.5 and 5 mg/kg/week), Intraperitonial, 5 weeks	Provided prophylactic effect against cardiac toxicity	El Shaer et al., 2017
Graphene oxide gold nanosheets (GO-Au)	chitosan-GO-Au scaffold	Rat, acute	5×2 mm scaffold	Improved the cardiac contractility and restored ventricular functions	Saravanan et al., 2018

**TABLE 3:** List of selective studies using organic-inorganic nanoparticles for the delivery of therapeutics for the repair of myocardial infracted heart tissue.

**Nanoparticles/size (nm)**	**Therapeutic agents**	**MI Model**	**Dose/administration route**	**Results**	**References**
DNAzyme-conjugated AuNPs/14± 3	Silence TNF-α	Rat, acute	100 μL, intramyocardial	Significant anti-inflammatory benefits and improved cardiac function	Somasuntharam et al., 2016
Organic-inorganic hybrid hollow mesoporous organosilica nanoparticles (HMONs)/20	Hepatocyte growth factor (HGF) gene-transfected BMMSCs	Rat, acute	2 × 10^6^ HGF gene-transfected BMMSCs, intramyocardial	Decreased apoptotic cardiomyocytes, reduced infarct scar size, relieved interstitial fibrosis, increased angiogenesis, and improved cardiac functions	Zhu et al., 2016
Complex of recombinant baculovirus and Tat/DNA nanoparticles/500	Angiopoietin-1 gene	Rat, acute	300 μL, intramyocardial	Increased capillary density, reduced infarct size and improved cardiac functions	Paul et al., 2011

Here, we review the studies done over the last 10 years that investigated the applications of different NP types for repairing cardiac tissue after myocardial infarction and also summarize treatment efficacies of different NP types. Furthermore, some of the advances, challenges, and future strategies in this field are also provided.

## Organic Nanoparticles

### Lipid-Based NPs

Typical lipid-based NP formulations (Figure 1A) include solid-lipid nanoparticles, nanostructured lipid carriers, lipid-drug conjugates, and nanoemulsions; all are primarily comprised of physiological lipid analogs with surfactants as stabilizers (Qi J. et al., 2017). According to the size of lipid-based nanoparticles, they are named as micelles (∼15 nm), liposomes (∼100 nm) or polymeric NPs (Paulis et al., 2012; Vinhas et al., 2017). Micelles consist of lipids and other amphiphilic artificial molecules that self-assemble in aqueous solution and form a monolayer with the hydrophobic phase inside that incorporates hydrophobic therapeutic agents (Katsuki et al., 2017). The enclosed space in the micelle is more confined than that in liposomes (Katsuki et al., 2017). Liposomes are heavily investigated in nanomedicine and are the first to get FDA approval for nanomedicine (Katsuki et al., 2017; Vinhas et al., 2017). Liposomes mainly consist of phospholipids that form bilayers with the aqueous phase inside, conferring superior biocompatibility to the liposomes (Katsuki et al., 2017). Polymeric nanoparticles, such as polylactic acid (PLA), polyglycolic acid (PGA), and poly lactic-co-glycolic acid (PLGA) are FDA-approved polymers. PLGA is a copolymer of PLA and PGA and is being tested for drug delivery systems for intractable diseases, including cardiovascular diseases (Pascual-Gil et al., 2017; Oduk et al., 2018).

Lipid NPs are broadly considered as promising candidates for the delivery of therapeutics in the infarcted heart. They possess morphology similar to that of cell membranes and can incorporate both lipophilic and hydrophilic substances (Saludas et al., 2018). They have successfully demonstrated the ability to deliver several biomaterials in the target tissue, such as low molecular weight drugs, imaging agents, peptides, proteins, and nucleic acids (Cheraghi et al., 2017). Paulis et al. (2012) reported that micelles are promising vehicles for the delivery of cardioprotective drugs, needed for the acute stage of MI, and also for the delivery of drugs that regulate infarct healing during the chronic stage of MI. On the other hand, liposomes are more suited for the delivery of pro-angiogenic drugs to the infarct microvasculature (Paulis et al., 2012).

### Dendrimers

Dendrimers (Figure 1B) are the smallest of all the nanocarriers and they have their multiple end groups that are appropriate for a high degree of link targeting or the active agents (Morgan et al., 2005). Dendrimers are dendritically expanded macromolecules with monodisperse structure consisting of a central core, branching interior and exterior functional groups (Morgan et al., 2005). Dendrimers possess the advantage of enhancing the binding capacity upon modification of their exterior surface with some ligands or antibodies for active targeting (Thomas et al., 2013); also, they can carry drugs with poor solubility (Singh et al., 2016). Xue et al. (2018) reported that cardiomyocyte apoptosis and infraction size were significantly reduced following single intravenous administration of dendrimer (15 μg), loaded with microRNA-1 inhibitor, in the acute mice MI model.

Selective studies using organic nanoparticles for the delivery of therapeutics to repair infarcted myocardial tissue were listed as Table 1. Paulis et al. (2012) reported that micelles are promising vehicles for the delivery of cardioprotective drugs, needed for the acute stage of MI, and also for the delivery of drugs that regulate infarct healing during the chronic stage of MI. On the other hand, liposomes are more suited for the delivery of pro-angiogenic drugs to the infarct microvasculature. However, to achieve cardiac protection after myocardial infarction, some therapeutic cargoes were required. So far, a large number of agents were loaded into nanoparticles targeted for different purposes. Recent studies have demonstrated that encapsulating ROS, Puerarin or Baicalin into micelles or lipids to reduce infarct size of the animals’ ischemic heart (Zhang et al., 2016; Dong et al., 2017; Vong et al., 2018). For cardiomyocyte apoptosis prevention, IGF1, liraglutide, Nitroxyl radical, Cyclosporine A, Pitavastatin or 2,2,6,6-tetramethyl piperidine-1-oxyl was embarked on lipid-based NPs and sent to the animals’ ischemic heart (Chang et al., 2013; Yin et al., 2014; Asanuma et al., 2017; Mao et al., 2017; Qi Q. et al., 2017). Intravenous injection of collapsin response mediator protein-2 (CRMP2) lipid with the size of 50 nm was shown fibrosis reducing in the mice chronic MI heart (Zhou et al., 2015). For vasculogenesis enhancement, VEGF, FGF1, Ang-1, stromal cell-derived factor-1 (SDF-1) or CCR2 was loaded in NPs and delivered to the ischemic myocardial tissue to stimulate angiogenesis (Paul et al., 2011; Lu et al., 2015; Oduk et al., 2018; Ding et al., 2020; Fan et al., 2020). Interestingly, Wang et al. (2018) recently reported that no statistically significant improvements in cardiac function and infarct size were detected in mice acute MI heart with the intravenous administration of CCR2 targeting-nanoparticles (micelles) vs. non-targeted micelles. Recently, nanoparticle delivery through intravenous injection with targeting peptides has merged has a promising strategy. Xue et al. (2018) reported an early targeting therapy for myocardial infarcted mouse through the tail vein with anti-miR-1 antisense oligonucleotide (AMO-1) loaded and myocardium-targeting dendrimer: PEGylated dendrigraft poly-L-lysine with angiotensin II type 1 receptor (AT1-PEG-DGL AMO-1). They found that AT1-PEG-DGL quickly accumulated in the MI heart during the desired early period, significantly outperforming the group without AT1 targeting. Apoptotic cell death in the infarct border zone was significantly decreased and the myocardial infarct size was reduced by 64.1% with a single IV injection as compared with that in MI group (Xue et al., 2018).

## Inorganic Nanoparticles

### Carbon-Based Nanoparticles

#### Carbon Nanotubes

Carbon nanotubes (CNT) (Figure 1C) are a subfamily of fullerenes and are composed of graphite sheets that are rolled up into tubular forms (Katsuki et al., 2017). As nano-carriers, they incorporate drugs in their inner space and present chemically modified external surfaces with biological molecules, such as nucleotides and proteins, to provide selective targeting (Katsuki et al., 2017). Based on their number of layers, carbon nanotubes are categorized as either single-walled or multi-walled (Sajid et al., 2016). The poor solubility of drugs, faster deactivation, and limited bioavailability can be addressed by using these carbon nanotubes which are preferentially used as drug carriers (Raphey et al., 2019). However, one of the major disadvantages of the CNT is the chance for their dissociation in biological fluids (Raphey et al., 2019). Nevertheless, carbon nanotube is a well-suited drug carrier for enhanced penetration in the cells and also for offering privileged drug actions (Zhang et al., 2011). Their unique optical, electrical, and mechanical properties make them a suitable candidate for potential therapeutic applications (Gorain et al., 2018). Moreover, a couple of studies have validated the promising potentials of CNT in cardiac tissue engineering, such as in the support of cardiomyocyte function and growth (Ahadian et al., 2017; Sun et al., 2017a) and acceleration of the gap junction formation (Martinelli et al., 2013; Shin et al., 2013). Aside these studies, other investigations have suggested that scaffold consisting of col-hydrogel and CNT could be promising injectable biomaterial to deliver drugs and cells for cardiac tissue regeneration in the infarcted myocardial tissues (Sun et al., 2017b; Gorain et al., 2018).

#### Graphene

The nanotechnology field is in constant research of novel materials that can be engineered for the precise, sensitive, and selective detection of biomarkers (Tang et al., 2020). Recently, the graphene-based family of materials has shown huge potential as their proposed biosensing applications have shown great diversity (Stankovich et al., 2006; Geim and Novoselov, 2007; Bitounis et al., 2013). The isolated two dimensional (2D) crystal structures composed of single atomic layers of graphite are called “graphene” (Figure 1D; Bitounis et al., 2013). In Novoselov et al. (2004) isolated and characterized a single sheet of graphene. Since then, research on graphene has been highly increasing and has attracted a deep interest in scientific fields (Bitounis et al., 2013; Paul et al., 2014). Feng et al. (2011) pioneered the successful use of graphene as a non-toxic nano-vehicle for efficient gene transfection. With all atoms exposed on its surface, graphene has an ultra-high surface area available for efficient loading of aromatic drug molecules via π-π stacking, providing a plethora of applications in drug delivery via stable complex formation and avoiding chemical conjugation (Sun et al., 2008; Zhang et al., 2010). Paul et al. (2014) reported that methacrylated gelatin hydrogel (GelMA) impregnated with functionalized graphene oxide (fGO) nanosheets, where the latter were complexed with pro-angiogenic human vascular endothelial growth factor plasmid DNA (pDNAVEGF), formed nanocomposite hydrogels (fGOVEGF/GelMA) that efficiently transfect the myocardial tissues and induce favorable therapeutic effects without invoking adverse cytotoxic effects. Nevertheless, adverse reactions induced by graphene-based materials on exposure will depend on multiple factors that need to be scrutinized (Bitounis et al., 2013). Therefore, clinical translation of graphene-based materials is still in its infancy, yet the field holds tremendous potential for the treatment of multiple diseases (Bitounis et al., 2013).

### Metal Nanoparticles

Nanogold, also called gold nanoparticles (GNPs) or colloidal gold (Figure 1E), has been actively investigated in a wide variety of biomedical applications (Zhou Y. et al., 2018). The unique physical and chemical properties, such as ease of bio-conjugation, excellent stability, superior security, and strong biocompatibility of many GNPs make them promising candidates in nanomedicine (Sperling et al., 2008).

Silver nanoparticles (AgNPs) (Figure 1F) have been developed as potent anti-microbial agents and have a multitude of applications, such as in toothpastes, bedding, water purification, and nursing bottles (Priyadarsini et al., 2018). After oral exposure, it is shown that about 18% of silver could be absorbed in humans (Bostan et al., 2016). Animal studies showed that AgNPs exposure will cause enhanced superoxide anion production and cause deleterious effects in cardiac tissues (Ebabe Elle et al., 2013; Lin et al., 2017; Xu et al., 2018). Thus, toxicity concerns of AgNPs have limited their effective translation for the cardiac tissue repair.

Cerium oxide (CeO_2_) nanoparticles (Figure 1G) have wide applications, such as in oxygen sensors and automotive catalytic converters (Niu et al., 2011). These nanoparticles are considered potent remedial options for the treatment of smoking-related diseases (El Shaer et al., 2017) since intravenous injection of these nanoparticles have shown a marked reduction in the myocardial oxidative stress and have also shown a significant reduction of the left ventricular dysfunction in the murine models of heart failure (Niu et al., 2011). The well-known mechanism underlying the action of these nanoparticles is attributed to their dual oxidation state, where the loss of oxygen and the reduction of Ce^4+^ to Ce^3+^ are accompanied by the creation of an oxygen vacancy (Niu et al., 2007).

Selective studies using inorganic nanoparticles for the delivery of therapeutics to repair infarcted myocardial tissue were listed as Table 2. Unlike the organic nanoparticles, the inorganic nanomaterials alone (without therapeutic agents loaded) could provide mechanical support even enhance cell electrical signaling in some conducting nanomaterials (Zhou J. et al., 2018). Zhou et al., created a conductive hydrogel by introducing graphene oxide (GO) nanoparticles into oligo(poly(ethylene glycol) fumarate) (OPF) hydrogels and delivered to the Sprague Dawley rats’ acute MI heart by peri-infarct intramyocardial injection. They found that injected OPF/GO hydrogels can not only provide mechanical support but also electric connection between normal cardiomyocytes and the myocardium in the scar via activating the canonical Wnt signaling pathway, thus upregulating the generation of Cx43 and gap junction-associated proteins (Zhou J. et al., 2018). However, inorganic nanoparticles loaded with potential therapeutic agents have been widely studied. Similar to the studies of organic nanoparticles, scholars mainly aim at oxidative stress-reducing, inflammation attenuation, cardiomyocyte apoptosis prevention, fibrosis reducing and vasculogenesis enhancement (Han et al., 2018; Sharma et al., 2018). Copper has shown the anti-inflammatory, anti-oxidant potential and cardioprotective effect. Sharma et al. treated treat the I/R rat with low dose copper nanoparticles (CuNP) (1 mg/kg/day, p.o., 4 weeks) and myocardial protection was detected like the reduction of oxidative stress, inflammatory cytokines and apoptosis through phosphorylate GSK-3β kinase pathways (Sharma et al., 2018). Gold nanoparticles (AuNPs) delivered intravenously (400 μg/kg/day, 14 consecutive days) may also improve myocardial injury after myocardial infarction in rats with the decrease of eNOs immunoreaction, Bcl-2 and collagen fibers (Ahmed et al., 2017). However, in another mouse acute MI model, AuNPs intravenous administration (100 μl/day, 7 days) accumulated in infarcted hearts, decreased infarction size, inhibited cardiac fibrosis but has no effect on apoptosis and hypertrophy (Tian et al., 2018). Inflammation attenuation was shown in mouse MI models intramyocardial injection of 50 μl interleukin-4 plasmid DNA-functionalized macrophage-targeting graphene oxide complex (MGC/IL-4 pDNA) via a reduction in intracellular ROS and developing M2 macrophage phenotypes in macrophages (Han et al., 2018). Similar to the organic nanoparticles, intramyocardial injection of a nano-complex of graphene oxide loaded with vascular endothelial growth factor-165 (VEGF) gene in the rat acute MI model shows significant infarct size reduction and capillary density enhancement (Paul et al., 2014). Further studies are needed to elucidate the long-term biocompatibility and safety of these inorganic nanoparticles.

## Organic-Inorganic Hybrid Nanoparticles

Recently, interests in the applications of various organic-inorganic hybrid nanoparticles (NPs) have risen tremendously. Hybrid NPs combine features of organic and inorganic building blocks and generate NPs with improved physicochemical properties, such as particle size and surface charge (Haque and Chowdhury, 2018). Hybrid organic-inorganic NPs hold great promise in overcoming the pitfalls being faced by existing inorganic materials in the delivery of therapeutics and contrast agents (Somasuntharam et al., 2016), such as unwanted interactions with serum proteins (particularly opsonins) and consequential removal from the circulation by macrophages of mononuclear phagocytic system, rapid renal clearance, prolonged body accumulation, and lack of targetability (Zhou and Zhang, 2019).

Magnetoliposomes (MLs) are composed of liposomes and magnetic NPs and are the first efficient hybrid liposome/NP systems produced for the drug delivery (Namdari et al., 2017). In this line, several experimental strategies have been investigated the potential scope of magnetic NPs to leverage the delivery of growth factors, cytokines, and biomolecules to the degenerating cardiac cells and tissues and enhance their regeneration (Allen and Cullis, 2013; Paul et al., 2014; Ottersbach et al., 2018).

Selective studies using organic-inorganic nanoparticles for the delivery of therapeutics to repair infarcted myocardial tissue were listed as Table 3. Somasuntharam et al. (2016) created deoxyribozyme (DNAzyme) functionalized AuNPs to catalytically silence tumor necrosis factor-α (TNF-α) as a potential therapeutic for acute myocardial infarction. After the intramyocardial injection, with the silencing of TNF-α, significant anti-inflammatory benefits, and cardiac function improvement were detected in the rat heart. With the same model, (Paul et al., 2011) design a new gene delivery method utilizing a self-assembled binary complex of negatively charged baculovirus (Bac) and positively charged endosomolytic histidine-rich Tat peptide/DNA nanoparticles (NP) together with Angiopoietin-1 (Ang-1) gene carried by. 3 weeks post intramyocardially delivery, cardiac function improvement, capillary density enhancement, and infarct sizes reduction were detected in Bac-NP(Ang1) compared to Bac(Ang1), NP(Ang1) and control groups due to enhanced myocardial Ang-1 expression at peri-infarct regions. Furthermore, (Zhu et al., 2016) designed and synthesized molecularly organic-inorganic hybrid hollow mesoporous organosilica nanoparticles (HMONs) for gene transfection (hepatocyte growth factor, HGF) in BMMSCs and subsequent *in vivo* cardiac repair. The fabricated organic-inorganic hybrid HMONs with large pore size represent a generalizable strategy to promote the ischemic myocardium therapeutic potential of HGF transfected BMMSCs including reduction of apoptotic cardiomyocytes, infarct scar size, and interstitial fibrosis while increasing angiogenesis (Zhu et al., 2016).

## Artificial DNA Nanostructures

The success of DNA nanotechnology lies in the artificially constructed special nanostructure design systems for DNA computing (Lee et al., 2016). DNA nanostructures, owing to their precise control over chemistry, size, and shape, provide vast opportunity to unfold the convoluted mass of information relating to nanoparticle-biological interactions (Lee et al., 2016). Drug delivery and therapeutics is considered as one of the most promising applications of the structural DNA nanotechnology (Ke et al., 2018). In this line, artificial nucleic acid nano-devices could be utilized to provide targeted drug delivery in the tissues upon sensing their environment (Singh et al., 2016). Moreover, several studies have proposed various DNA nanostructures and strategies to load, deliver, and release biomolecular drugs for cardiac therapy (Paul et al., 2011).

## Comparison of the Nanoparticles as for Ischemic Myocardium Repair

Nanoparticles of different types (for example, inorganic, organic and hybrid) designed to target ischemic cardiac cells are promising candidates for the treatment of myocardial infarction. Organic nanoparticles are offering numerous advantages which embrace the simplicity of their preparation from well-understood biodegradable, biocompatible polymers, and their high stability in biological fluids during storage (Virlan et al., 2016). Since the emergence of nanotechnology in the past decades, polymeric materials such as poly (d-lactic acid), polyethylene glycol (PEG) and poly lacticco-glycolic acid (PLGA) have emerged as a major class of biodegradable and controlled release systems for delivering biomolecules/proteins to the plaque site (Fredman et al., 2015; Kamaly et al., 2016).

The use of inorganic nanoparticles for applications in drug delivery presents a wide array of advantages, which include: (1) Ease of functionality with a range of surface and conjugation chemistries; (2) High payload loadings; (3) Tunable degradation rates; and (4) Enhanced penetration into tissue (Pandey and Dahiya, 2016). Magnetic nanoparticles were shown to accelerate the expression of critical gap junction proteins (for example, connexin 43) in cardiomyoblasts. These new cells demonstrated higher levels of both engraftment capacity and desirable paracrine factors compared with conventional therapeutic cells, thus significantly enhanced heart function and reduced scar size when delivered into the peri-infarcted area in rats (Han et al., 2015). Superparamagnetic iron oxide nanoparticles, with biocompatibility and capacity for simultaneous imaging and targeting, have emerged as the major particles for enhancing the engraftment of therapeutic cells in heart tissue. However, it was recently revealed that these nanoparticles increase tumor-associated macrophage activation (Zanganeh et al., 2016).

Intensive studies have thoroughly probed the toxicities of a wide range of nanoparticles (organic, inorganic, and polymeric) in different types of cells and organs. However, the cardiotoxicity of nanoparticles has been poorly investigated, and data are still limited to a few types of nanoparticle including metal oxides, silver, and carbon (Bostan et al., 2016). The main limiting issue for the design of safe and efficient nanoparticles for the treatment of ischemic heart disease is the lack of a deep understanding of the biological identity of nanoparticles. To accelerate the clinical translation of nanoparticles for use in cardiac nanotechnology, their biological identities must be precisely assessed and reported (Mahmoudi et al., 2017). The advantage and disadvantage of each NP category were summarized in Table 4.

**TABLE 4:** Advantages and disadvantages of different nanoparticles for the delivery of therapeutics to repair infarcted myocardial tissue.

**Type**	**Advantages**	**Disadvantages**	**References**
Lipid-based NPs	Increased penetration and/or permeation, Biocompatible and biodegradable nature, Easy and scalable production process, Increased drug solubility, Possibility of specific follicular targeting, Good stability during storage period.	Loss of high amounts of drug, Lack of robust controlled drug release, Burst drug release may induce toxic effects, Macrophage drug clearance (rapid clearance).	Ghasemiyeh and Mohammadi-Samani, 2018
Dendrimers	Ease of fabrication, targeting ability, potential for repeat administration, low immune response and precise controllability of the functionality.	Great batch-to-batch variability and deterioration, the attachment of multiple molecules can often result in a population of conjugates with a wide distribution of the number of ligands.	Pearson et al., 2012
Carbon Nanotubes	Extremely small and lightweight, Resources required are plentiful, Resistant to temperature changes, Highly flexible and elastic, and Improve conductive mechanical properties.	Still don’t understand how they work, Difficult to work with, Toxic qualities, Lack of solubility in most solvents, Susceptibility to oxidative environments, Difficulty in maintaining high quality and minimal impurities.	Pitroda et al., 2016
Metal Nanoparticles	Strong plasma absorption, Biological system imaging, Determine chemical information on metallic nanoscale substrate.	Instability, impurity, explosion and safety concerns (Toxicity),	Harish et al., 2018

## Clinical Application

A large number of patents, pertinent to the invention of cardiovascular biomaterials, have been filed in the past decade. Importantly, an invention of the UV-crosslinkable gelatin methacrylate-based cardiac patch, impregnated with gold nano-rods, was recently patented (US20170143871 A1). The patent describes about patch that exhibits high surface area and electrical conductivity. Another recent invention describes a combination of gold nano-wires and engineered scaffolds for controlling the cellular function through electronic circuits (US20170072109 A1). Furthermore, a new strategy of nanoparticle-stem cell electrostatically conjugates for post-infarction treatment was patented in Japan (JP5495215 B2). A preparation method of nanomagnetic particles for the detection and treatment of coronary heart diseases was patented in China (CN102085380A). This method can be used for preparing magnetic nanoparticles for mid-late-stage treatment of coronary heart diseases; and good repairing and treatment effects on coronary heart diseases can be achieved. In another patent, a solid lipid nanoparticle of Gelan Xinning Ruanjiaonang (Chinese traditional medicine) for treating coronary heart disease was created and beneficial for clinical application (CN103027981B). The treatment effect of Gelan Xinning Ruanjiaonang for coronary heart disease is significantly improved with the solid lipid nanoparticle included. Despite several patented technologies for cardiovascular therapeutics, only a few have entered into the clinical trials, due to the stringent regulatory requirements (Lakshmanan and Maulik, 2018). Fortunately, some of the targeted drug nanocarriers for cardiac therapies successfully passed clinical trials (Galagudza et al., 2010) and are already commercially available (Chong et al., 2014). For instance, one of the clinical strategies that has been practiced since long for inducing angiogenesis in the ischemic tissues includes intramuscular transplantation of the micro-bubbles and causing ultrasound-mediated microbubble destruction for the delivery of entrapped bone marrow-derived mononuclear cells (BM-MNCs) to provide tissue regeneration (Tateishi-Yuyama et al., 2002).

## Current State-Of-The-Art Nanotechnology Used in Cardiac Therapy and Future Perspectives

Despite initial encouraging results from nanotechnology-based cardiac protection, poor retention time, efficacy, side effects or off-target effects of the delivered NPs remain major obstacles for efficient myocardial regeneration (Chang et al., 2013; Somasuntharam et al., 2013; Yin et al., 2014; Zhou et al., 2015). Several delivery strategies like intracoronary, intramyocardial, or intravenous have been applied for cardiac repair. Traditionally, NPs were injected via intracoronary or intramyocardial route and they rely on open heart surgery (Chang et al., 2013; Somasuntharam et al., 2013). Most adverse effects were observed they were delivered either intravenously or orally (Yin et al., 2014; Zhou et al., 2015). So far, no strategy has been proven to replace the transmural scar tissue in the chronic infarcted heart tissues. However, the current state-of-the-art nanoparticle technologies have emerged as one of the most promising strategies for myocardial repair (Awada et al., 2016). With the application of heart targeted agents, efficacy could be highly improved, while lowering the adverse effects by delivering NPs by intravenous route (Nguyen J. et al., 2015; Ferreira et al., 2016). The active targeting agents include MMP-2 and MMP-9 targeting peptides (Nguyen M. M. et al., 2015), which may results in long-term retention at the site of infarction. The atrial natriuretic peptide (ANP) is a circulating cardiac hormone produced physiologically, which belongs to the natriuretic peptide family and has been shown to have cardioprotective properties through cGMP-dependent signaling involving guanylyl cyclase A (GC-A) receptors (Potter et al., 2009). Peptide CSTSMLKAC and CRSWNKADNRSC are cyclic structures and have shown alone selective targeting to the ischemic heart (Kanki et al., 2011; Ferreira et al., 2016). Furthermore, heart homing agents make oral administration or inhalation administration an alternative and promising approach (Miragoli et al., 2018; Sharma et al., 2018).

**FIGURE 2:**
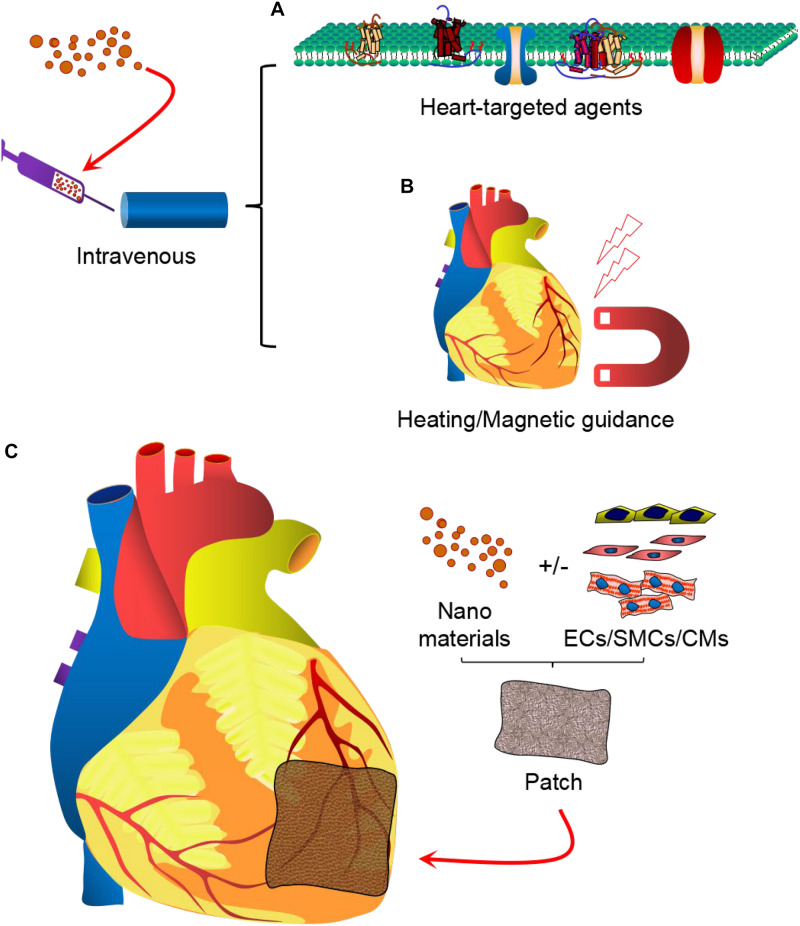
Nanoparticle-based therapy for cardiovascular diseases. Therapeutic strategies may involve intravenous delivery of drug-loaded nanomaterials, modified with cardiac tissue-targeting moieties **(A)** or guided with non-invasive heating or magnetic field **(B)**. Furthermore, cardiac patch may be applied locally in the infarct region to provide simultaneous delivery of the nanoparticles and induced pluripotent stem cell-derived cardiovascular cells (cardiomyocytes, endothelial cells, and smooth muscle cells) **(C)** to provide robust myocardial tissue regeneration and functional improvements.

The majority of the *in vivo* studies have shown the great potential of the nanoparticle systems in improving the function and tissue regeneration of the infarcted myocardium. However, further improvement in the homing and delivery of these nanoparticles and their therapeutic effects, respectively, to the target tissues can be achieved via decoration of these nanoparticle systems using heart-targeting active molecules (Figure 2A) or using non-invasive physical cues (Figure 2B). Furthermore, future clinical strategy may involve the application of cardiac patch that not only delivers the therapeutic agents, but its scaffolding effect provides optimal mechanical support to the failing heart and also replaces lost cells and tissues with induced pluripotent stem cell-derived cardiovascular cells, such as cardiomyocytes, endothelial cells and smooth muscle cells (Figure 2C). Thus, it is expected that advances in drug therapy, nanomedicine, cell-therapy, and material science will provide robust functional improvement and tissue restoration in patients with myocardial infarction in the near future.

## Author Contributions

CF, JJ, and WZ wrote the manuscript. FL, BX, MK, JY, and WZ, revised the manuscript. All authors approved the submission and publication of the manuscript.

## Conflict of Interest

The authors declare that the research was conducted in the absence of any commercial or financial relationships that could be construed as a potential conflict of interest.
